# Negative versus positive priming: When are distractors inhibited?

**DOI:** 10.16910/jemr.10.2.6

**Published:** 2017-05-20

**Authors:** Stefan Van der Stigchel, Martijn Meeter

**Affiliations:** Utrecht University, Netherlands; VU University Amsterdam, Netherlands

**Keywords:** Intertrial priming, saccades, negative priming, positive priming, visual search

## Abstract

Visual attention is guided by the history of selections in previous trials, an effect usually referred to as intertrial priming. The aim of the present study was to investigate whether such priming in visual search is due to a strengthening of the target signal, or the suppression of the distractor signal. In two experiments, we examined the deviation of saccade endpoints in situations in which the target and distractors were presented in relative close proximity. We found both negative and positive priming, irrespective of whether the repeating feature was relevant or irrelevant. This finding is in contrast to previous results with this paradigm, based on which we concluded that visual priming is strictly the result of boosting perceptual target signals. Based on the differences between these experiments, we conclude that the number of distractors is essential in observing negative priming. We propose that negative priming is solely observed when multiple distractors result in either strong inhibition of distractor features, or strong adaptation to them. Whereas positive priming seems to be a robust mechanism, negative priming is only present if there are multiple distractors.

## Introduction

Visual attention is guided not only by top-down and bottom-up factors, such as task demands or visual saliency, but also by the history of previous selections. Attention is deployed with higher likelihood to stimuli that share features with those previously attended to. In visual search, this is most evident from the effects of recent search trials on the current one: Finding a target on a current trial is more easy when it is similar to the target on previous trials, compared to when the target changed identity ( 7, 9, 10, 12 ).

Such priming has been attributed to attentional selection being biased towards previous target features, which aids the search for subsequent targets when they share those features, compared to when they do not ( 5, 11 ). For example, Becker, Ansorge and Horstmann ( 2 ) investigated search in a task that required participants to make an eye movement to the target. They found that the first eye movement to a target was faster when the target-defining feature was repeated than when it changed. This, they argued, shows that selection itself was facilitated by priming, and not some post-selection process. Although such a finding convincingly argues against a post-selection locus of priming, it is currently unclear what is changed by a previous trial that speeds attention selection: does the target repetition result in a strengthening of the target signal in a saliency map ( 1, 11 ) or does the repetition of the distractors result in a suppression of the distractor signals ( 9 )?

To investigate this question, we recently developed an eye movement paradigm to disentangle effects of an enhanced target effect from that of suppressed distractors ( 6 ). In this experiment, we placed two elements in relatively close proximity. This is known to result in the global effect, a tendency for saccades to land in between the two elements, instead of on one of them ( 4, 14, 17, 18 ). The global effect is most pronounced for saccades with a short latency, as these eye movements are hypothesized to be dominated by bottom-up visual information. For these saccades, the eye movement can be considered an averaged saccade program towards the two elements, resulting in a saccade vector pointing to the intermediate location. Interestingly, the saccade endpoint is known to reflect the strength of the individual signals: a saccade will land closer to the element that evokes the stronger signal, for example because it is bigger or brighter ( 4 ) or matches the content of visual working memory ( 15 ). The global effect paradigm therefore allows one to dissociate the strength of the individual signals of target and distractor in a visual search task.

In our previous study, we varied the colors of the target and the distractor from trial to trial. The target was defined by shape. Even though color was thus an irrelevant dimension in this task, color repetition still resulted in priming: Saccades landed closer to the target when it had the color of the target on the previous trial, suggesting that priming enhances target color signals. Even more convincingly, when the current distractor received the color of the previous target, the increased strength of the signal associated with the previous target color transferred to it, resulting in saccades that were directed more towards the distractor. These effects were even observed for the fastest eye movements, initiated some 130 ms after the presentation of the two elements. At this timescale, in the range of express saccades, eye movements are hypothesized to be initiated based purely on the visual information evoked by the onsets and without any influence of top-down information ( 13 ). This finding ruled out any post-selection explanation, as such an explanation would result in modulations restricted to saccades with a longer latency. For instance, the N2pc, a physiological indicator of the allocation of attention, is typically observed around 200 to 300 ms after a search display is presented ( 3 ), showing that these fast saccades might indeed be initiated before attention is deployed. Based on these findings, we concluded that visual priming is at least partly the result of boosting perceptual target signals.

Distractor color repetition, on the other hand, had no effect. This was somewhat surprising, as previous studies have shown that distractor repetition speeds search even when target features are not repeated ( 6 ), suggesting that some form of distractor suppression might also be involved in priming in visual search. One difference between previous studies and ours is that in ours search was quite limited: there were only two elements that were both placed in the same part of the display. Although this setup was well-suited to scrutinize the contribution of the individual signals in priming, it did not allow examining priming in its most extensively investigated form: priming of pop-out (PoP) (9). In PoP, the target has a unique feature that makes it pop out of the display, and that is repeated or not on the subsequent trial. Target feature repetition typically results in an even faster detection than when the unique feature is not repeated. Furthermore, color was task-irrelevant in our previous study, which could contribute to the lack of an effect of distractor color repetition. It could very well be that an effect of distractor color repetition is solely observed when color is relevant to distinguish the target from distractors. Such a finding would indicate that target boosting is a robust effect which occurs independently of relevance, whereas distractor suppression only occurs for the relevant feature active in the search template.

To investigate the strength of target and distractor signals in popout displays, we generated global effect saccades in displays in which the target popped out from multiple distractors. In Experiment 1, a uniquely colored target was presented together with five distractors that all shared the same color, whereas in Experiment 2, we presented displays consisting of one target and two distractors. We varied the colors of target and distractors from trial to trial. In line with our previous study, we expected a boost of the target signal (i.e. a deviation towards the distractors, when these distractors had the color of the target of the previous trial). If distractor suppression occurs in PoP, we expect saccade endpoints to deviate more strongly towards the distractor in the conditions in which the target color matches the distractor color of the previous trial.

## Methods Experiment 1

Thirteen naive participants (on average 24 years old, range 22-30; 4 male) participated in the experiment. Informed consent was obtained prior to the study in accordance with the guidelines of the Helsinki Declaration. Eye movements were recorded by an Eyelink1000 system.

**Figure 1:**
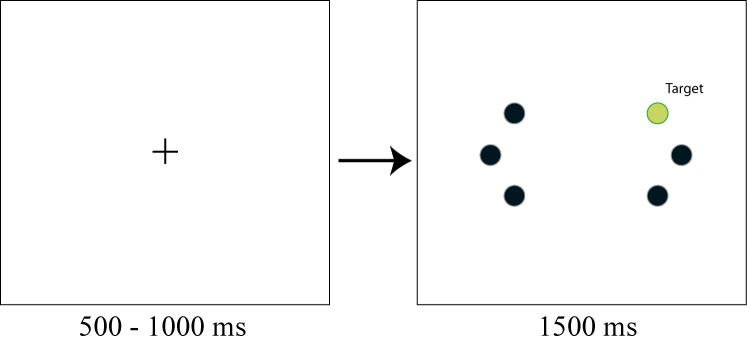
Displays used in Experiment 1. Participants first saw a fixation cross that remained on the screen for a variable interval. This cross disappeared at the same time that six circles appeared in the periphery. One of these circles was a color singleton and the task of the participant was to make a saccade to the singleton as fast and accurately as possible.

Participants viewed a display containing a gray cross (1.0 x 1.0º) on a black background in the centre of the display, which was used as fixation point. The fixation point was removed after a random interval of 500-1000 ms. Subsequently, six filled circles were presented (diameter: .67º). The distance from the central fixation point to the stimuli was 7.7°. The six circles were presented at fixed locations at mirrored locations in the left and right visual fields. For each visual field, one circle was presented in the same horizontal plane as the fixation point. The two other circles were presented on the same imaginary circle and were positioned 22.5° clockwise and 22.5° counterclockwise (see Figure 1). The target circle could be presented at one of the six possible locations. The target circle had a different color than the other five circles, which all had the same color. The target display was presented for 1500 ms. Afterwards all objects were removed from the display.

All elements could have one of six, approximately isoluminant colors (around 20 cd/m2): blue, green, yellow, brown, red, and purple. There were four, equally likely, repetition conditions: - Both repeated: Target and distractor both had the same color as on the previous trial. - Distractor becomes target: The target had the color of the distractor on the previous trial, whereas the distractor had a new color. - Target becomes distractor: The distractor had the color of the target on the previous trial, whereas the target had a new color. - Both new: Target and distractor had different colors that were new compared to the previous trial. This condition functioned as the implicit benchmark condition, against which the effect of the other conditions was measured.

Participants were instructed to fixate on the central fixation cross and to move their eyes to the target on the monitor as quickly as possible. The sequence of trials was randomized. When condition determined that either the target or the distractor condition should change, the new color was chosen randomly from those not used on the preceding trial. The experiment consisted of 720 experimental trials and 24 practice trials.

## Data analysis

### Saccade endpoint

Only trials in which the target was presented 22.5° clockwise and 22.5° counterclockwise from the horizontal plane were analyzed, as the endpoints in these trials could be interpreted in terms of a deviation towards or away from the other elements. Because saccades can also land away from a distractor (19), such an interpretation is not possible for the trials in which the target was presented at the horizontal plane. Because saccade averaging only occurs when target and distractor are closely aligned (20), the circle at the horizontal plane (which is closest to the target) will be referred to as the 'distractor'. Distractors in the opposite visual field were placed outside of the global effect zone (i.e. around 20° in polar coordinates, 20).

Saccadic endpoint was computed as a proportion of the angle between the target and the distractor, which we will refer to as endpoint deviation, Φ. The target was used as a null reference, whereas the distractor had a deviation score of +1. Saccades with a Φ below 0.5 landed closer to the target than to the distractor, while the opposite was true for deviations above 0.5. Saccades with a Φ below -0.5 or above 1.5 (meaning that the saccade did not land in between the two stimuli by a 50% margin) were excluded from the analysis; this was the case for, on average, less than 4 saccades per participant.

To examine the time-course of effects, each participant’s saccades were in each condition rank ordered from shortest to longest latency, and partitioned into five equalsized latency bins. The first bin contained the 20% fastest saccades that the participant made in a certain condition, whereas the last contained the slowest saccades. For each participant, the average saccade endpoint per condition and per latency bin was then calculated. Bin was treated as a linear factor in all of the analyses.

### Saccade latency

Saccade latency was defined as the interval between target onset and the initiation of the saccadic eye movement. Trials with a saccadic latency lower than 80 ms (anticipatory saccades) or higher than 800 ms were excluded (too slow saccades). Of all saccades in Experiment 1, 0.4% were discarded because they were too fast (<80 ms), and none because they were too slow (>800 ms).

## Results

### Saccadic reaction time

A within-subject ANOVA with as factors condition and bin showed a main effect of both condition, F(3,36)=9.57, p<.001, partial η²=.44, and bin, F(1,12)=117.6, p<.001, partial η²=.91. Planned deviance contrasts with Both New as a reference condition (using Bonferroni correction for multiple comparisons) showed that SRT was lower for the Both Repeated condition (m=206, sd=6.1) than for the Both New condition (m=208, sd=6.8), F(1,12)=10.48,p=.021, partial η²=.466. There was no difference between the Both New and the Target becomes Distractor condition (m=208, sd=6.8), F(1,12)=6.62,p=.072, partial η²=.355. SRTs in the Distractor Becomes Target condition (m=214, sd=7.5) were slower than those in the Both New condition, F(1,12)=18.95,p=.003, partial η²=.612. In lieu of a traditional interaction between bin and condition, we compared the slopes, as a function of bin, in the first three conditions to that of the Both New condition. None of those comparisons were significant after Bonferroni correction for multiple comparison (p>.052).

**Figure 2:**
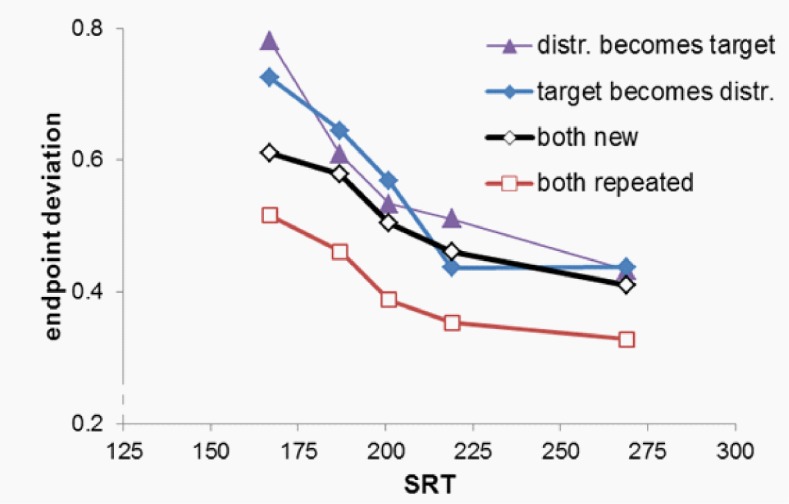
Saccade endpoint deviation as a function of condition and the mean saccadic reaction time (SRT), in five latency bins (bin midpoints were, for the figure, averaged over all conditions). A value of 0 indicates a saccade on the target, and a value of 1 indicates a saccade to the distractor. Intermediate values indicate endpoints between the two.

### Endpoint deviation

Figure 2 shows saccadic endpoint, rescaled to the interval 0 (target position) to 1 (position of the closest distractor). A within-subject ANOVA with as factors condition and bin showed a main effect of both condition, F(3,36)= 38.7, p<.001, partial η²=.76, and bin, F(1,12)=63.43, p<.001, partial η²=.84, and an interaction between these two factors (see below). Planned contrasts showed that saccades deviated more to the target in the Both Repeated than in the Both New condition, F(1,12)= 84.5, p<.001, partial η²=.88, while saccades deviated more strongly towards the distractor in both the Target becomes Distractor condition, F(1,12)=30.2, p<.001, partial η²=.72, and in the Distractor Becomes Target condition, F(1,12)=25.8, p<.001, partial η²=.68. Moreover, endpoint deviation changed more steeply with bin in the Target becomes Distractor than in the Both New condition, F(1,12)=8.14, p=.039, partial η²=.41. There was no difference in the effect of bin between the Both New condition and either the Both Repeated or the Distractor becomes Target condition (p>.11).

## Discussion

The results of Experiment 1 showed clear priming due to colors used on the previous trial. When the target and the distractor both had the same color as on the previous trial ('Both repeated'), saccadic reaction times were lower and the saccade endpoint shifted towards the target. Because both the target and the distractor are repeated, it is unclear whether this was due to a target boost or a distractor suppression, or due to a combination of both. These effects could be disentangled using the conditions in which only the target or distractor color was repeated. In the condition in which the target had the color of the distractor on the previous trial ('Distractor becomes target'), the saccade endpoint deviated away from the target and saccadic reaction times were increased, showing negative priming due to a suppression of the distractor color. Positive priming was observed in the condition in which the distractor had the color of the target on the previous trial ('Target becomes distractor'). Also here, we observed a shift of the saccade endpoint towards the distractor, but the explanation here is a boost of the target signal of the previous trial, resulting in a stronger distractor signal when the distractor now had the color of the target of the previous trial. Importantly, these effects were not restricted to either faster or slower saccades, showing that these effects do not reflect post-selection processes.

Thus, both negative and positive priming were observed in the present experiment, in contrast to our previous study in which only positive priming was observed ( 6 ). In the current study, color was a relevant feature, because participants were instructed to saccade to the color singleton, whereas participants searched for the shape singleton in our previous experiment. Besides this difference, there was an additional difference between the two studies. In our previous study, there was more uncertainty about the possible locations of the elements compared to the current Experiment 1. Whereas the elements were presented around the horizontal meridian in Experiment 1, the elements could be presented anywhere on a circle around fixation in our previous study. To investigate whether the presence of negative color priming was due to the relevance of color or due to the lower uncertainty of the possible target and distractor locations, we performed an additional experiment in which the elements were presented on an unpredictable location around fixation. Moreover, we investigated the relevance of color by having the participants search for a color singleton in one block, and for a constant target shape in the other.

## Methods Experiment 2

Sixteen naive participants (on average 26 years old, range 21-39; 6 male) participated in the experiment.

After removal of the fixation point, three elements were presented: two filled circles (diameter: .67º) and one filled square (1.0 x 1.0º). The centre element was randomly positioned on one of eight equidistant axes (polar coordinates: 22.5°, 67.5°, etc.). The other two elements were presented 22.5° clockwise and counterclockwise from the centre element. The square was presented at one of these two peripheral locations ('shape singleton'). The other peripheral location was occupied by a circle with a different color from the other two elements ('color singleton'). The centre element was always a circle with the same color as the square. Therefore, the shape singleton and the color singleton were always presented at the peripheral of the three locations. The target display was presented for 1500 ms. Afterwards all objects were removed from the display.

**Figure 3:**
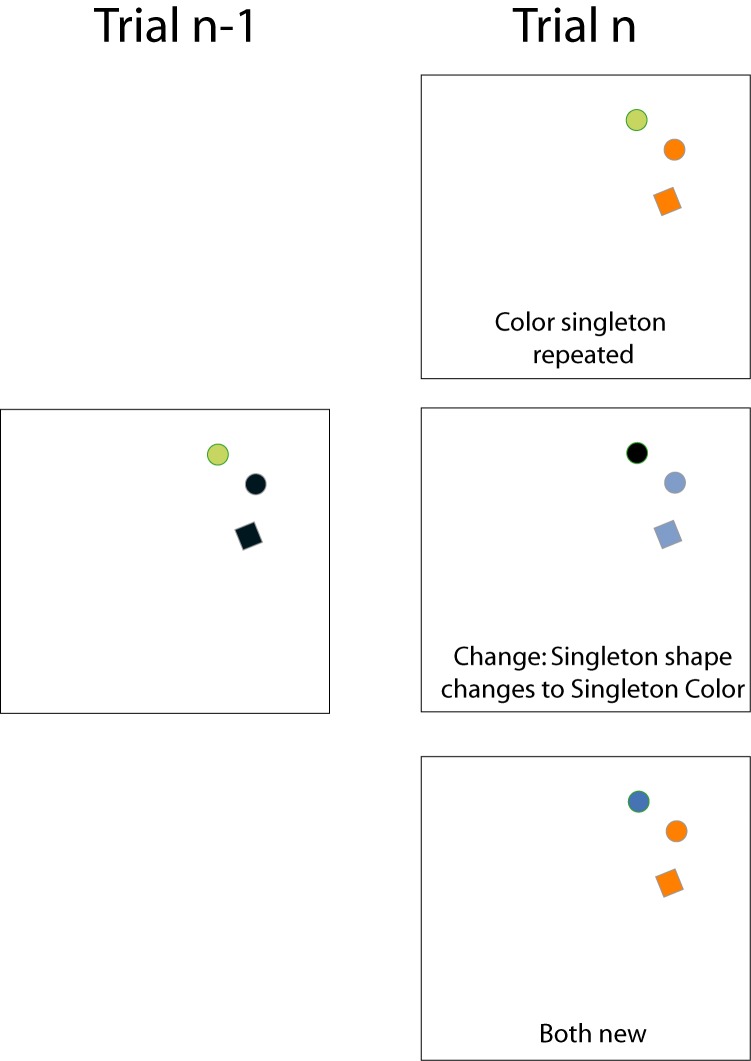
The three conditions used in Experiment 2 for a given trial n-1. Note that we used six colors and all combinations of colors were possible in the experiment.

**Figure 4:**
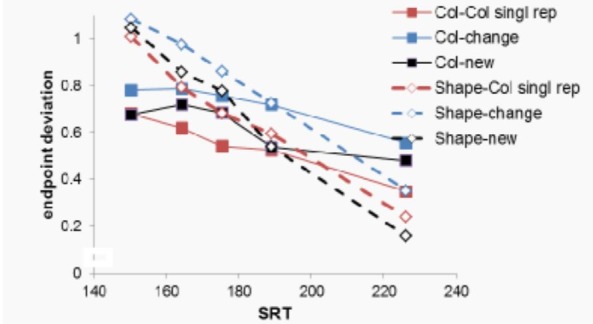
Saccade endpoint deviation as a function of condition and the mean saccadic reaction time (SRT), in five latency bins (bin midpoints were, for the figure, averaged over all conditions). A value of 0 indicates a saccade on the target, and a value of 1 indicates a saccade to the distractor. Intermediate values indicate endpoints between the two.

There were three, equally likely, repetition conditions (see Figure 3). - Color singleton repeated: The color singleton had the same color as on the previous trial, but the other two elements shared a new color. - Singleton shape changes to Singleton color (‘Change’ in Figure 4): The color singleton had the color of the other two elements on the previous trial, whereas the other two elements shared a new color. - Both new: All elements had different colors that were new compared to the previous trial, but the color of the color singleton differed from the color of the other two elements. This condition functioned as the implicit benchmark condition, against which the effect of the other conditions was measured. In this experiment, the colors of the target and distractors were therefore never both repeated or swapped from trial to trial. We had two blocks which were presented in counterbalanced order. In the color block, participants were instructed to move their eyes to the color singleton on the monitor as quickly as possible. In the shape block, participants were instructed to move their eyes to the shape singleton. The sequence of trials was randomized. Each block consisted of 720 experimental trials and 24 practice trials. In Experiment 2, 0.6% were discarded because they were too fast, and less than 1 saccade per participant because they were too slow.

## Results

### Saccadic reaction time

A within-subject ANOVA with as factors task, condition and bin showed no effects, F<1.36, p>.26, except that of bin, F(1,15)=100.96, p<.001, partial η²=.87.

### Endpoint deviation

Figure 4 shows saccadic endpoint, rescaled to the interval 0 (target position) to 1 (position of the closest distractor). A within-subject ANOVA with as factors task (Shape vs Color), condition and bin showed a main effect of condition, F(2,30)= 23.54, p<.001, partial η²=.61, and of bin, F(1,15)=33.67.1, p<.001, partial η²=.69, but not of task, F(1, 15)= 1.04, p=.324, partial η²=.065.

There was a significant interaction between bin and task in that bin affected saccadic endpoint differently as a function of task, F(1, 8)= 15.49, p=.001, partial η²=.508, with steeper slopes in the Shape task than in the Color task. We performed a separate ANOVA only on the Color task data to ascertain that endpoint were nonetheless closer to the target for later bins in this task, which was indeed the case, F(1,15)= 11.43, p=.004, partial η²=.432. There were no differences in how bin affected saccadic endpoint as a function of either condition (F<1.49, p>.24) or an interaction of condition and task, (F<2.14, p>.125).

The observation that task did not interact with condition shows that the main effect of condition was observed for both the color and the shape task. Planned contrasts for this main effect of condition, using Both New as reference condition and Bonferroni correction for multiple comparison, showed that saccadic endpoint was closer to distractors in the Singleton shape changes to Singleton color condition than in the Both New condition, F(1,15)=23,5, p<.001, partial η²=.61. For the color task, this is evidence for distractor inhibition, as the color of the target was associated with the distractor on the previous trial. For the shape task, however, this is evidence for target boosting, as the color of the distractor was associated with the target on the previous trial.

There was a trend for the endpoint to be closer to the target in the Color Singleton Repeated condition relative to the Both New condition, F(1,15)=4.5, p=.051, partial η²=.23. For the color task, this is evidence for target boosting, as the color of the target was associated with the target on the previous trial. For the shape task, however, this is evidence for distractor inhibition, as the color of the distractor was associated with the distractor on the previous trial.

## General discussion

The aim of the present study was to investigate whether priming effects in visual search are due to a strengthening of the target signal or suppression of the distractor signal. To disentangle these possibilities, we made use of the finding that the deviation of the endpoint of a saccade towards a certain element reflects the relative strength of this element ( 4, 18 ). We therefore examined the deviation of saccade endpoints in situations in which the target and distractors were presented in relative close proximity. The results of Experiment 1 showed that the saccade endpoint shifted towards the uniquely colored target when the color of the target was repeated in the presence of five distractors. Additional analyses revealed that both positive and negative priming contributed to this shift towards the target. These results were replicated in Experiment 2, in which there was more uncertainty regarding the possible locations of the different elements. In Experiment 2, we also changed the search template to investigate whether the lack of distractor suppression in our previous study was due to the relevance of the changing feature. We again found both negative and positive priming, irrespective of whether the repeating feature was relevant or irrelevant. These effects were not restricted to either faster or slower saccades, showing that these effects do not reflect post-selection processes.

When compared to the results of our previous study, these findings provide insight in the flexible nature of negative priming. Note that in our previous study, we only observed positive priming and found no evidence for negative priming ( 6 ). To explain this result, we attributed the lack of negative priming to the fact that the repeating feature was irrelevant for the search task. In this view, target boosting is a robust process, whereas distractor suppression only occurs for the relevant feature of the search template. The present findings provide clear evidence against the idea that the relevance of the repeating feature is the crucial factor that determines whether negative priming is observed. Although we indeed observed both positive and negative priming when the repeating feature was task-relevant in Experiment 1 and 2, negative priming was clearly present in the condition in Experiment 2 in which the repeating feature was task-irrelevant.

One additional possibility for the inconsistency between the different findings is that in our previous study, there was uncertainty about the possible locations of the elements. In contrast, the elements in the current Experiment 1 were always presented around the horizontal meridian. It could be negative priming is only observed in case there is a certain amount of uncertainty regarding the spatial lay-out of the search task. We therefore introduced spatial uncertainty in Experiment 2 but still found evidence for negative priming, indicating that the uncertainty of the spatial lay-out does not play a crucial role in determining the presence of negative priming.

The only remaining difference between the previous experiment and the experiments reported in the present study is the number of distractors that were presented with the target. Whereas there was only one distractor in our previous experiment, the number of distractors in the current study was either five (Experiment 1) or two (Experiment 2). The idea that the number of distractors is essential in observing negative priming is confirmed by the observation that one of the conditions in Experiment 2 (i.e. the shape condition) is simply a replication of our previous experiment with the addition of one distractor. The additional distractor determined whether or not negative priming is observed.

Two explanations for this pattern of data come to mind. First, it may be that the presence of additional distractors increases the strength of inhibition required to make an eye movement to the target. When multiple distractors are inhibited to correctly select the target, the feature associated with the distractors may become associated with this increased inhibition relative to when there is only one distractor. When relatively little inhibition was required on the previous trial (i.e. in case of a single distractor), this inhibition may not carry over to the subsequent trial, and no negative priming is then observed on the subsequent trial. An alternative explanation is in line with a recently proposed computational model of intertrial priming. Kruijne and Meeter (8) suggested that positive priming results from the intrinsic reward of honing in on a target, while negative priming results from passive adaptation to visual stimuli – whether they are targets or distractors. When there are many distractors in the display, adaptation will mainly be to distractors, while when there is just one target and one distractor in the display, adaptation will be indiscriminate. This would result in the data pattern observed here and in our previous study ( 6 ), with negligible negative priming when there is one distractor, and stronger negative priming when there are multiple distractors in the display. On the basis of the current evidence, both explanations are equally plausible.

To sum up, we propose that negative priming is solely observed when multiple distractors result in either strong inhibition of distractor features, or strong adaptation to them. Whereas positive priming seems to be a robust mechanism, negative priming is only present if there are multiple distractors.

### Ethics and Conflict of Interest

The authors declares that the contents of the article are in agreement with the ethics described in http://biblio.unibe.ch/portale/elibrary/BOP/jemr/ethics.html and that there is no conflict of interest regarding the publication of this paper.

### Acknowledgements

This research was funded by grant 452-13-008 from NWO (Netherlands organization for Scientific Research) to SvdS.
